# A novel cold-adapted and highly salt-tolerant esterase from *Alkalibacterium* sp. SL3 from the sediment of a soda lake

**DOI:** 10.1038/srep19494

**Published:** 2016-02-26

**Authors:** Guozeng Wang, Qiaohuang Wang, Xianju Lin, Tzi Bun Ng, Renxiang Yan, Juan Lin, Xiuyun Ye

**Affiliations:** 1 College of Biological Science and Engineering, Fuzhou University, Fuzhou 350108, P. R. China; 2 Fujian Key Laboratory of Marine Enzyme Engineering, Fuzhou 350002, P.R. China; 3 School of Biomedical Sciences, Faculty of Medicine, The Chinese University of Hong Kong, Hong Kong, China

## Abstract

A novel esterase gene (*estSL3*) was cloned from the *Alkalibacterium* sp. SL3, which was isolated from the sediment of soda lake Dabusu. The 636-bp full-length gene encodes a polypeptide of 211 amino acid residues that is closely related with putative GDSL family lipases from *Alkalibacterium* and *Enterococcus*. The gene was successfully expressed in *E. coli*, and the recombinant protein (rEstSL3) was purified to electrophoretic homogeneity and characterized. rEstSL3 exhibited the highest activity towards *p*NP-acetate and had no activity towards *p*NP-esters with acyl chains longer than C8. The enzyme was highly cold-adapted, showing an apparent temperature optimum of 30 °C and remaining approximately 70% of the activity at 0 °C. It was active and stable over the pH range from 7 to 10, and highly salt-tolerant up to 5 M NaCl. Moreover, rEstSL3 was strongly resistant to most tested metal ions, chemical reagents, detergents and organic solvents. Amino acid composition analysis indicated that EstSL3 had fewer proline residues, hydrogen bonds and salt bridges than mesophilic and thermophilic counterparts, but more acidic amino acids and less hydrophobic amino acids when compared with other salt-tolerant esterases. The cold active, salt-tolerant and chemical-resistant properties make it a promising enzyme for basic research and industrial applications.

Esterases (EC 3.1.1.1) and lipases (EC 3.1.1.3) are a class of hydrolytic enzymes that catalyze the hydrolysis and transesterification of fatty acid esters^1^. These enzymes are grouped into the family of serine hydrolases and share some similar structural and functional characteristics, including a conserved catalytic triad (Ser-Asp/Glu-His), a consensus sequence (Gly-x-Ser-x-Gly) around the active residue serine^2^, an α/β hydrolase fold and cofactor-independent activity^3^. Esterases differ from lipases mainly in terms of the kinetics and substrate specificity^4^. Esterases have classical Michaelis-Menten kinetics and prefer water-soluble short-chain fatty acid esters (C < 10), whereas lipases prefer water-insoluble substrates, typically long-chain triglycerides (C > 10). Based on the amino-acid sequences and fundamental biological properties, bacterial esterases and lipases have been grouped into eight families^5^. A new subfamily of hydrolytic/lipolytic enzymes designated as SGNH-hydrolase superfamily or subfamily has been proposed based on the oxyanion structure^6^. Enzymes in this subfamily typically have a GDSL motif including the active serine that is located near the N terminus, and exhibit multifunctional properties and regiospecificity.

Esterases and lipases are widely distributed in nature and can be found in animals, plants and microorganisms. Microbial esterases and lipases have been extensively studied due to their great potential for applications in the medical and pharmaceutical industries, detergents, synthesis of fine chemicals, and bioremediation^2^,^7^. The majority of esterases and lipases that are currently used in industry are obtained from mesophilic fungi or bacteria. However, those from extremophiles, also known as extremozymes, are attracting much attention due to their excellent performance under extreme physico-chemical conditions and superiority over their counterparts for industrial applications^8^,^9^. Lipolytic enzymes from thermophiles and hyperthermophiles, psychrophiles, halophiles, alkalophiles/acidophiles, and solvent-resistant microorganisms have been studied by using culture-dependent and -independent methods^8^,^10^. Among them, lipolytic enzymes from (halo)alkaliphiles are of interest for their application potentials in laundry detergents, finishing of fabrics, and pulp and paper industries^8^.

Soda lake represents a natural sodium carbonate/bicarbonate-buffered system and is one of the most stable alkaline environments^11^. It is characterized by high alkalinity (pH from 9.0 to 12.0) and moderately to extremely high salinity (up to saturation), and provides a unique habitat to harbor a rich diversity of (halo)alkaliphilic bacteria and archaea^11^,^12^. These microorganisms are excellent sources of novel genes, enzymes and biomolecules with biotechnological potentials. For example, two industrial cellulases from the Gram-positive isolates of soda lake Kenyan have been used in textile and laundry processes^13^; and alkaline proteases, lipase, amylases, chitinases and caseinases have also been reported from the microorganisms isolated from soda lakes^14^. Until recently, few studies on the esterases and lipases from soda lake microorganisms have been reported^15^,^16^.

In this study, a novel esterase gene (*estSL3*) was cloned from *Alkalibacterium* sp. SL3, which was isolated from the sediment of soda lake Dabusu. Sequence analysis suggested that EstSL3 belongs to the GDSL family and represents a member of the subfamily of SGNH hydrolases. The enzyme produced in *E. coli* showed cold-adapted, highly salt-tolerant and chemical/detergent/solvent-resistant properties. These properties make EstSL3 a good candidate for basic research and wide industrial applications.

## Results

### Strain identification

Based on the BLASTn analysis, the 16S rDNA sequence of strain SL3 (1515 bp) showed a nucleotide identity of 99.5% with that of *Alkalibacterium* sp. E-119 (FJ764767), 99.2% with *A. pelagium* strain NBRC 103242 (NR_114241), and 99.1% with *A. pelagium* strain T143-1-1 (NR_041574). Thus, strain SL3 was classified into the genus *Alkalibacterium*. The distance tree created by the neighbor-joining method also revealed the same classification (data not shown).

### Gene cloning and sequence analysis of EstSL3

DNA fragments amplified through TAIL-PCR were assembled with the 3′ region, and an ORF of 636 bp, starting with ATG and terminating with TAA, was identified. The full-length gene (*estSL3*) encoded a polypeptide of 211 amino acid residues. No signal peptide was predicted. Its calculated molecular mass and theoretical isoelectric point were estimated to be 24.04 kDa and 5.28, respectively.

**Figure 1:**
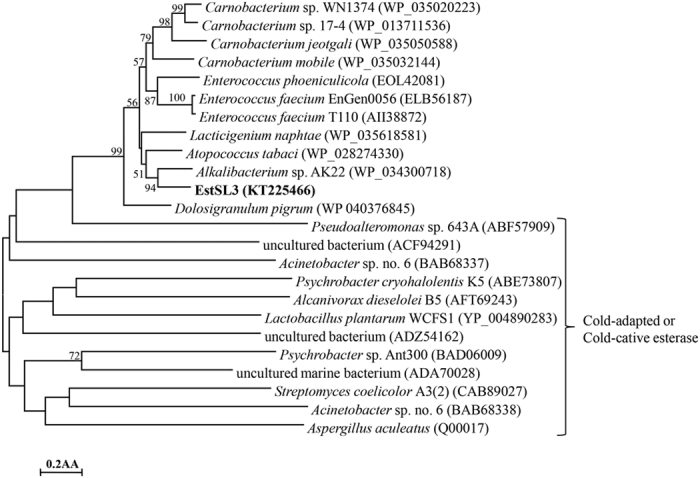
Phylogenetic tree of the amino acid sequences of EstSL3 and its close homologs. The tree was constructed using the neighbor-joining method (MEGA 4.0). Bootstrap values (n = 1,000 replicates) are reported as percentages. The scale bar represents the number of changes per amino acid position. The sequence accession numbers are given at the end of each species name.

The deduced amino acid sequence of *estSL3* showed the highest identity (69%) with a putative GDSL family lipase from *Alkalibacterium* sp. AK22 (WP_034300718), followed by the putative GDSL family lipases from *Atopococcus tabaci* (WP_028274330, 65% identity) and *Lacticigenium naphtae* (WP_035618581, 58% identity). A phylogenetic tree was constructed based on the amino acid sequences of EstSL3, its closest homologs and cold-adapted esterases retrieved from GenBank database. High bootstrap values separated these esterases into five major groups (Fig. 1). EstSL3 was closely related to the putative esterases from *Alkalibacterium* sp. AK22 (WP_034300718), *A. tabaci* (WP_028274330) and *L. naphtae* (WP_035618581), but was distant from other known cold-adapted esterases with functional verification.

Based on the multiple sequence alignment of EstSL3 and eight other esterases, five conserved regions of the GDSL family^6^ were identified (Fig. S1). Three putative catalytic residues, Ser^15^, Asp^189^, and His^192^, were located in the conserved regions, and two residues, Gly^59^ and Asn^88^, might form an oxyanion hole with Ser^15^. These results indicated that EstSL3 belongs to the SGNH hydrolase subfamily.

**Figure 2:**
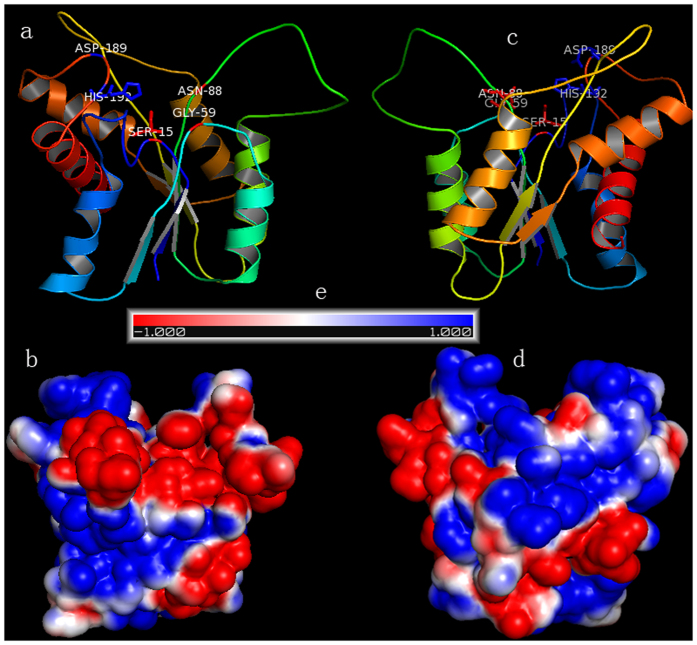
Structure and surface electrostatic potential analysis of EstSL3. (**a)** Modeled EstSL3 constructed by I-TASSER with 3w7vA used as the template. (**b**) The surface electrostatic potential of EstSL3 obtained by Pymol and APBS plugin. (**c**) The 180° rotated view of (**a**). (**d**) The 180° rotated view of (**b**). (**e**) The negative and positive electrostatic potentials are indicated by *blue* and *red*, respectively.

Modeled EstSL3 had a typical structure of SGNH hydrolases (Fig. 2), consisting of a single domain of five-stranded, parallel β-sheets, three helices at the convex side and two helices at the concave side of the sheet, and a short helix at the domain edge for ornament^6^. The catalytic triad of Ser^15^, Asp^189^ and His^192^ was located in a groove, and Gly^59^ and Asn^88^ together with Ser^15^ formed the oxyanion hole.

### Expression and purification of rEstSL3

The gene fragment coding for the protein was expressed in *E. coli* BL21 (DE3). After induction with 0.5 mM IPTG at 30 °C for 12 h, significant esterase activity was detected after cell lysis. The crude enzyme was purified to electrophoretic homogeneity by Ni-affinity chromatography (Fig. S2). The purified rEstSL3 migrated as a single band of approximately 25 kDa on SDS-PAGE, which was identical to the calculated value (24.04 kDa). Three internal peptides obtained from LC–ESI-MS/MS, LTVLNRGIGGDSLKDLK, TDARVILMESFVLPYPKR and VGWRNDLDK, matched the deduced amino acid sequence of EstSL3 (Fig. S1), confirming that the purified enzyme was indeed EstSL3.

### Enzyme characterization

Among the tested *p*NP-esters, EstSL3 exhibited the highest activity towards *p*NP acetate (256.86 ± 3.52 U · mg^–1^) and weak activity to *p*NP-butyrate and -caproate (33.39 ± 2.55 U · mg^–1^ and 23.63 ± 2.72 U · mg^–1^, respectively). No activity was detected against *p*NP-esters with the side chain longer than C8.

**Figure 3:**
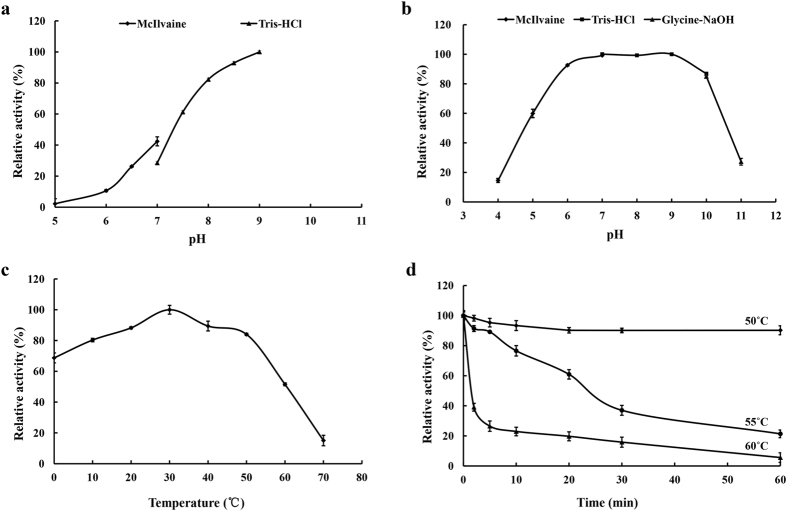
Enzymatic properties of purified rEstSL3. (**a**) Effect of pH on EstSL3 activity. Activities at various pHs were assayed at 37 °C for 5 min. (**b**) pH stability of EstSL3. Residual activities after incubation at various pHs for 1 h at 37 °C were assayed at pH 9.0 and 30 °C for 5 min. (**c**) Effect of temperature on EstSL3 activity in Tris-HCl buffer (pH 9.0). (**d**) Thermostability of EstSL3. Residual activity was assayed at pH 9.0 and 30 °C for 5 min after pre-incubation at 50 °C, 55 °C or 60 °C for different periods of time. The data are shown as means ± SD (n = 3).

When assayed at 30 °C and using *p*NP-acetate as the substrate, purified rEstSL3 showed the highest activity at pH 9.0 among the pH tested, and remained 60% of the maximum activity between pH 7.5 and 9.0 (Fig. 3a). The rEstSL3 activity at pH higher than 9.0 was not tested due to the high self-degradation of *p*NP-acetate at alkaline pHs. The enzyme was stable over the neutral to alkaline pH range, retaining more than 80% of the initial activity after incubation in buffers ranging from pH 6.0 to 10.0 at 37 °C for 1 h (Fig. 3b). The thermal activity of purified rEstSL3 was apparently optimal at 30 °C when assayed at pH 9.0, and the enzyme retained greater than 50% of the maximum activity from 0 to 60 °C (Fig. 3c). At 0 °C, it exhibited 68% of the maximal activity. Without substrate, the enzyme was stable at 50 °C for more than 60 min, whereas at 55 °C and 60 °C, the half-lives of the enzyme were approximately 25 min and 2 min (Fig. 3d), respectively.

Using *p*NP-acetate as the substrate, the *K*_*m*_, *V*_*max*_, and *k*_cat_ values of EstSL3 were 0.15 ± 0.01 mM, 769.23 ± 5.68 μmol · mg^−1^ · min^−1^ and 307.69 s^−1^, respectively.

The effect of various metal ions and chemcial reagents on rEstSL3 activity was investigated (Table 1). The activity of the purified rEstSL3 was enhanced by Li^+^, Na^+^, K^+^, Mg^2+^, Ni^2+^, Ca^2+^, β-mercaptoethanol and EDTA at both tested concentrations (1 and 5 mM), but inhibited by Ag^+^, Fe^2+^, Cu^2+^, Mn^2+^, Hg^2+^, Co^2+^, Zn^2+^, Cr^3+^ and Pb^2+^ at higher or both concentrations.

rEstSL3 resistance against various detergents and organic solvents was also determined as shown in Table 2. As for the detergents we tested, the rEstSL3 activity was inhibited by tween-20 at 1–10% (v/v). In contrast, it was higly resisitent to tween-80, Triton X-100 and SDS, retaining more than 60% of the initial activity. On the other hand, The rEstSL3 activity was sensitive to all tested solvents. It was strongly inhibited by butanol, isobutanol, isoamyl, acetone and chloroform at concentrations of 20% (v/v), and slightly inhibited by methanol, ethanol, propanol, *n*-hexane, glycerol and acetonitrile.

**Figure 4:**
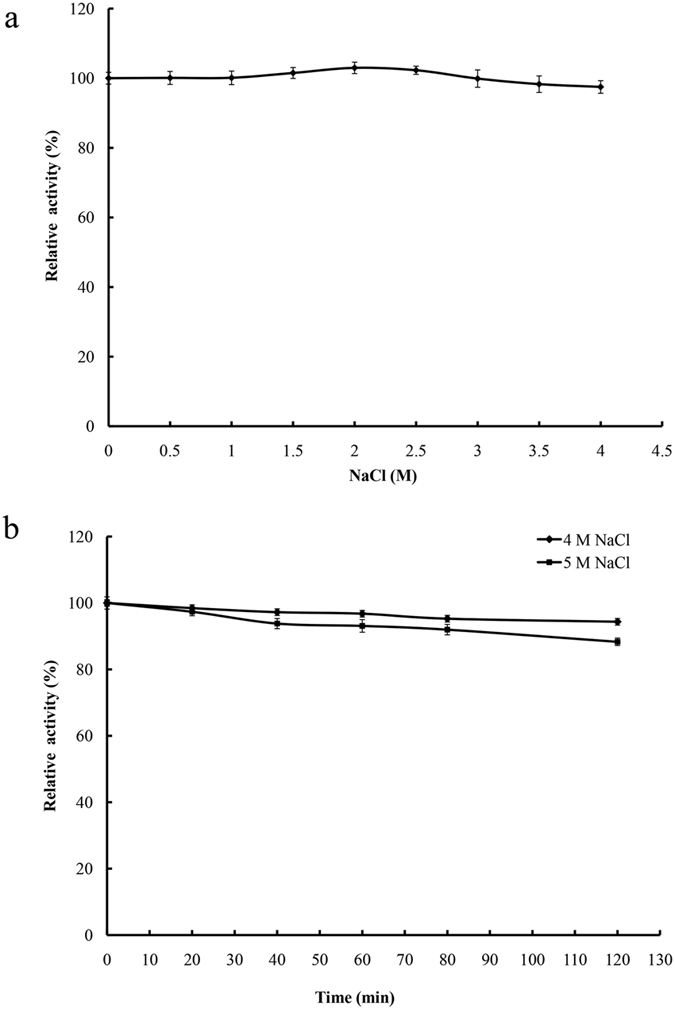
Effect of NaCl on rEstSL3 activity and stability. (**a**) Effect of different concentrations of NaCl on the activity of rEstSL3. (**b**) rEstSL3 stability in the presence of 4 M NaCl. The data are shown as means ± SD (n = 3).

Purified rEstSL3 showed the highest activity in the presence of 2 M NaCl and retained greater than 98% esterase activity in the presence of 0.5–4.0 M NaCl (Fig. 4a). Moreover, purified rEstSL3 showed strong tolerance to high concentrations of NaCl, retaining more than 87% esterase activity after 2-h incubation with 5 M NaCl at 37 °C and pH 9.0 (Fig. 4b).

## Discussion

Lipolytic enzymes from extremophiles have been attracting more and more attention due to their great application potentials in various industries^8^. Soda lake represent unique, saline and alkaline niche to harbor a microbial community that adapts to these extreme conditions^11^. Most of the microorganisms isolated from soda lakes have been described as halophiles and alkaliphiles, and diverse alkaline and salt-tolerant enzymes have been reported from them^12^. The lake Dabusu is a typical soda lake that has a high salinity and alkalinity^17^. Our previous studies have revealed the rich diversity of xylanase genes in the metagenome of soda lake Dabusu^18^, the source of thermophilic and salt- and alkaline-tolerant xylanase^19^. In this study, a novel esterase gene, *estSL3*, was cloned from *Alkalibacterium* sp.SL3, a strain isolated from the sediment of the lake Dabusu. EstSL3 has low similarities with known sequences, which are all putative lipases of GDSL family or hypothetical proteins without functional verification. Multiple sequence alignment of EstSL3 and eight close homologs (Fig. S1) suggested that EstSL3 could be classified into the SGNH subfamily that is characterized with broad substrate specificity and regiospecificity^6^. However, when EstSL3 was produced in *E. coli*, it exhibited a relatively narrow substrate spectrum. It showed the highest activity towards *p*NP-acetate and low or no activity towards *p*NP-esters with side chains longer than C2. Considering the difference between lipase and esterase, EstSL3 represents an esterase instead of a true lipase. To the best of our knowledge, this is the first report of an esterase from the genus *Alkalibacterium.*

Cold-adapted enzymes usually have low temperature optima and retain high activity at 0 °C^20^,^21^. rEstSL3 demonstrated the typical characteristics of cold-adapted enzymes, including a temperature optimum of 30 °C and 68% activity even at 0 °C (Fig. 3). Over the past few years, a number of cold-adapted esterases have been cloned and characterized from cultured microorganisms^22^,^23^,^24^,^25^ or by using metagenomic method^26^,^27^,^28^,^29^. Phylogenetic analysis (Fig. 1) and sequence alignment (Fig. S1) indicated that EstSL3 is distantly related to those reported cold-adapted or cold-active esterases and has some novelty. Except EstPc from *Psychrobacter cryohalolentis* K5^T^ 22, EstB from *Alcanivorax dieselolei* B-5(T)^25^ and Lp_2631 from *Lactobacillus plantarum*^30^, EstSL3 showed greater or comparable performance under low-temperature conditions to other characterized counterparts, including Est10 and Est11 from psychrotrophic *Psychrobacter pacificensis*^23^,^24^, Est97 from an Arctic intertidal metagenomic library^27^ and EstF from a deep-sea metagenomic library^26^. Although rEstSL3 owned the cold-adapted properties, it also had better thermostability than most cold-adapted esterases at high temperatures (50 °C). These characteristics may widen the application range of EstSL3 to the pharmaceutical, agricultural and chemical industries and so on.

Compared with mesophilic or thermophilic counterparts, cold-adapted enzymes usually employ several mechanisms to confer the enzymes high flexibility and catalytic efficiency at low temperatures. These factors include but are not limited to more glycine residues, fewer proline residues, a lower Arg/Arg + Lys ratio and a declined number of disulfide bridges and fewer weak interactions such as salt bridges, aromatic interactions and hydrogen bonds^20^,^21^. In the case of EstSL3, several characteristics adapting to cold-environment can be observed (Table 3). Firstly, it has fewer proline residues than cold active esterases EstPc^22^ and Est97 27, mesophilic esterase Axe2 31 and thermophilic esterase EstA^32^. Secondly, EstSL3 has fewer salt bridges. We found that all the cold-active esterases (EstSL3, Lip1Pc and Est97) have fewer salt bridges than mesophilic Axe2 and thermophilic EstA, and EstSL3 has even fewer salt bridges than cold-adapted Lip1Pc and Est97. Thirdly, fewer hydrogen bonds were found in EstSL3. The hydrogen bond percentage of EstSL3 is much lower than that of mesophilic Axe2, thermophilic EstA, and cold-active Lip1Pc and Est97. All of these factors in combination might lead to the enhanced flexibility but relatively increased stability of the structure of EstSL3 and consequently have a key role in maintaining its high catalytic activity at low temperature and great thermostability at high temperature.

Enzymes from bacteria isolated from soda lakes are usually capable of functioning at high pH and possibly high salt concentrations^12^. Although the activity of rEstSL3 at pH higher than 9 was not assayed due to the self-degradation of *p*NP-acetate at extreme alkaline pHs, EstSL3 could be regarded as an alkaline esterase based on the results we obtained (Fig. 3). Another noteworthy characteristic of rEstSL3 is the stability with increased NaCl concentration (Fig. 4). It is confirmed that an increase in the number of charged amino acids, especially acidic residues at the protein surface, can confer halophilic proteins enhanced activity at high salt concentrations^33^. We found that charged amino acids constitute about 30.3% (64/211) of EstSL3, with higher percentage of acidic residues (16.6%) than basic residues (13.7%). EstSL3 also has higher percentage of acidic residues when compared with other salt-tolerant esterases (Table 4). The surface electrostatic potential analysis suggested that the charged amino acids of EstSL3 are distributed on the surface of the protein (Fig. 2). Moreover, EstSL3 has lower percentage of hydrophobic amino acids. These factors might make EstSL3 to form a solvation shell that keeps the protein surface hydrated and thus highly tolerant to salt.

In addition, the effects of metal ions, detergents and organic solvents on rEstSL3 activity (Tables 1 and 2) were also studied. EstSL3 is resistant to most tested chemicals, including Li^+^, Na^+^, K^+^, Ni ^2+^, Mg^2+^, Ca^2+^, β-mercaptoethanol, EDTA, and low concentrations of tween-20, tween-80, Triton X-100, methanol, ethanol, propanol, Acetonitrile, glycerol and *n*-hexane. Most of the reported cold-active esterases are strongly inhibited by SDS, an anionic detergent that causes strong protein denaturation. For example, Est10 from *P. pacificensis* only retained 2.8% activity in the presence of 1% SDS^23^ while the EstPc was completely inhibited by 0.05% SDS^22^. In contrast, rEstSL3 retained 68.5% activity at the presence of 1% SDS, and had greater SDS-resistance over other cold-active esterases.

In conclusion, a novel esterase gene *estSL3* was cloned from a soda lake isolate, *Alkalibacterium* sp. SL3, and successfully expressed in *E. coli*. rEstSL3 is a cold-adapted, highly salt-tolerant enzyme with greater thermostability over most cold-active esterases. Moreover, the enzyme was slightly activated by several metal ions and detergents and showed tolerance towards SDS and organic solvents. All these enzymatic properties make EstSL3 a good candidate for basic research and broad industrial applications.

## Materials and Methods

### Strains, vectors and chemicals

Kits for genomic DNA isolation, DNA purification and plasmid isolation were purchased from Omega (Norcross, GA, USA). *E. coli* DH5α and the pMD 18-T vector (TaKaRa, Otsu, Japan) were used for gene cloning and sequencing, respectively. Restriction endonucleases, T4 DNA ligase, DNA polymerase and dNTPs were purchased from New England Biolabs (Ipswich, MA, USA). Vector pET-28a(+) (Novagen, San Diego, CA, USA) and *E. coli* BL21 (DE3) (TaKaRa) were used for gene expression. Nickel-NTA agarose (Qiagen, Valencia, CA, USA) was used to purify the His6-tagged protein. The substrates *p*NP acetate (C2), *p*NP butyrate (C4), *p*NP caproate (C6), *p*NP caprylate (C8), *p*NP caprate (C10), *p*NP myristate (C14), *p*NP palmitate (C16) were purchased from Sigma (St. Louis, MO, USA). Isopropyl-β-D-1-thiogalactopyranoside (IPTG) was purchased from Amresco (Solon, OH, USA). All other chemicals were of analytical grade and commercially available.

### Microorganism isolation

The sediment sample was collected from soda lake Dabusu, which is located in the southwest of Qian’an County, Jilin Province, China. The lake Dabusu has a salinity of 62.34 g L^−1^ to 347.34 g L^−1^ and a pH of 10 to 11^17^. Strain SL3 was isolated from the sediment sample as described by our previous study^19^. The taxon of the strain was identified by the 16S rDNA sequence PCR-amplified using primers 27F and 1492R (Table S1).

### Gene cloning of the full-length esterase gene (estSL3)

Genomic DNA was extracted from strain SL3 using the Omega genomic DNA isolation kit following the manufacturer’s instructions. The 3′ end of the esterase gene was obtained when we amplified the flanking regions of a xylanase gene by using thermal asymmetric interlaced (TAIL)-PCR^34^. Then three specific primers were designed to obtain the flanking region of 5′ end (Table S1). The PCR products were excised, purified, and ligated into vector pMD 18-T. The recombinant vector was then transformed into *E. coli* DH5α and sequenced by Invitrogen (Carlsbad, CA, USA). The full-length esterase gene was designated as *estSL3*.

### Sequence and phylogenetic analysis

The open reading frame (ORF) was identified by using the Vector NTI 10.3 (InforMax, Gaithersburg, MD, USA). The signal peptide sequence was predicted using SignalP (http://www.cbs.dtu.dk/services/SignalP/). The DNA and protein sequence similarities were assessed by using the BLASTn and BLASTp programs (http://www.ncbi.nlm.nih.gov/BLAST/), respectively. Multiple sequence alignments were performed with ClustalW (http://www.ebi.ac.uk/Tools/clustalw2/). A phylogenetic tree, including deduced EstSL3 and its closest homologs, was constructed using the neighbor-joining (NJ) algorithm in MEGA 4.0 35. Confidence for the tree topology was estimated using the bootstrap values based on 1,000 replicates.

### Putative structure analysis

The protein sequence of EstSL3 was submitted to I-TASSER^36^ (http://zhanglab.ccmb.med.umich.edu/I-TASSER/), which represents one of the best CASP^37^ wining *ab initio* protein folding and three-dimensional (3D) structure prediction servers. The acetylxylan esterase Axe2^31^ (PDB: 3w7v) from *Geobacillus stearothermophilus* was identified as a homologous template for EstSL3 modeling with the global sequence identity of 35%. Based on the estimated RMSD value and TM-score of 2.8 ± 2.1 Å and 0.90 ± 0.06, respectively, EstSL3 was classified as an ‘Easy’ target by I-TASSER and the predicted model is therefore reliable^38^. The predicted model and surface electrostatic potential were visualized via Pymol with the assistance of APBS plugin. Prediction of disulfide bridges, salt bridges (distances <3.2 Å), and hydrogen bonds were performed as described by Zhou *et al.*^39^. Two cold-adapted esterases LipPc^22^ and Est97^27^, a mesophilic esterase Axe2^31^ as well as a thermophilic esterase EstA^32^ were selected for comparison with EstSL3.

### Expression and purification of EstSL3 in *E. coli*

The full-length gene of *estSL3* was amplified by PCR using the expression primers (Table S1), and cloned into the *Nco*I-*Hind*III site of pET-28a (+). The recombinant plasmid, pET-*estSL3*, was transformed into *E. coli* BL21 (DE3) competent cells. Positive transformants harboring the recombinant plasmid (pET-*estSL3*) were identified by PCR and further confirmed by DNA sequencing. The cells were grown in LB medium containing 100 μg mL^−1^ of ampicillin at 37 °C to an A_600_ of 0.6. Protein expression was induced by addition of IPTG at a final concentration of 0.5 mM at 30 °C for 12 h. Esterase activities of the cell pellet were assayed as described below.

To purify the His-tagged recombinant protein (rEstSL3), the cells were harvested by centrifugation (12,000 × *g*, 4 °C for 10 min) and washed with sterile distilled water. The cells were then resuspended in sterilized ice-cold buffer (20 mM Tris-HCl, 0.5 M NaCl, pH 7.6) and disrupted by sonication (6 s, 160 W) on ice. The crude enzyme was collected (12,000 × *g* for 10 min at 4 °C) and loaded onto a Ni^2+^-NTA agarose gel column. The purified enzyme was washed with a linear imidazole gradient of 20–300 mM in Tris-HCl buffer (20 mM Tris-HCl, 500 mM NaCl, pH 7.6).

Sodium dodecyl sulfate-polyacrylamide gel electrophoresis (SDS-PAGE) was used to determine the purity and apparent molecular mass of rEstSL3. The protein concentration was determined by the Bradford method^40^, using bovine serum albumin as a standard. The identity of the purified enzyme was verified by liquid chromatography-electrospray ionization-tandem mass spectrometry (LC-ESI-MS/MS).

### Enzyme assay

Esterase activity assay was performed with *p*NP-esters as the substrates, and the production of *p*-nitrophenol was measured at 405 nm^41^. Reactions containing 0.1 mL of enzyme solution, 0.1 mL of 10 mM substrate and 1.8 mL of 50 mM Tris-HCl (pH 9.0) were incubated at 30 °C for 5 min. One unit (U) of esterase activity was defined as the amount of enzyme that released 1 μmol of *p*-nitrophenol per minute. The extinction coefficients of *p*-nitrophenol were measured at different pHs (ranged from 3,400 to 19,600 M^–1 ^cm^–1^) and used to correct the results. All reactions were performed in triplicate. The controls were reaction systems with addition of thermo-inactivated EstSL3.

### Biochemical characterization

The substrate specificity of purified rEstSL3 was determined in 50 mM Tris-HCl buffer (pH 9.0) containing 1 mM of *p*NP-esters (C2–C16) under standard conditions (30 °C and 5 min). The stock substrate solutions (10 mM) were prepared by dissolving *p*NP-esters in pure acetonitrile.

The optimal pH for esterase activity of the purified rEstSL3 was determined at 30 °C in buffers with pH ranging from 4.0 to 9.0. The stability of purified rEstSL3 at different pH values was estimated by incubating the enzyme solution in various buffers at 37 °C for 1 h without substrate. The remaining activity was measured in Tris-HCl buffer (pH 9.0) at 30 °C for 5 min. The initial activity of rEstSL3 was set as 100%. The buffers used were McIlvaine buffer (0.2 M Na_2_HPO_4_, 0.1 M citric acid) for pH 4.0–8.0, 0.1 M Tris-HCl for pH 8.0–9.0 and 0.1 M glycine-NaOH for pH 9.0–12.0.

The optimal temperature for purified rEstSL3 activity was determined over the range of 0–70 °C in Tris-HCl buffer (pH 9.0). Thermostability of rEstSL3 was determined by measuring the residual activities after pre-incubation of the enzyme in Tris-HCl buffer (pH 9.0) at 50, 55 and 60 °C for various periods.

The *K*_m_, *V*_max_, and *k*_cat_ values of rEstSL3 were determined in Tris-HCl buffer (pH 9.0) containing 0.05–5 mM *p*NP-acetate at 30 °C for 5 min. The *K*_m_ and *V*_max_ were determined from a Lineweaver-Burk plot using the non-linear regression computer program GraFit (Erithacus, Horley, Surrey, UK).

To investigate the effects of different metal ions on the purified rEstSL3 activity, the enzyme activities were measured at 30 °C in Tris-HCl buffer (pH 9.0) containing 1 or 5 mM (final concentration) of KCl, CaCl_2_, CoCl_2_, NiSO_4_, CuSO_4_, MgSO_4_, FeSO_4_, FeCl_3_, MnSO_4_, ZnSO_4_, Pb(CH_3_COO)_2_, AgCl, HgCl_2,_ β-mercaptoethanol and EDTA.

The effects of various detergents (tween-20, tween-80, SDS, and Triton X-100) and organic solvents (methanol, ethanol, propanol, butanol, isobutanol, isoamylol, acetone, *n*-hexane, glycerol, chloroform and acetonitrile) on the purified rEstSL3 activity were evaluated by adding different concentrations of detergents (1–10%, v/v) and organic solvents (10–20%, v/v). The enzyme activity without any addition was defined as 100%.

The effect of NaCl on the purified rEstSL3 activity was determined at 30 °C in Tris-HCl buffer (pH 9.0) containing 0.5–4.0 M NaCl. To examine its resistance to salt, rEstSL3 was incubated with 4 M of NaCl at 37 °C for 1 h, and the residual enzyme activities were measured.

### Nucleotide sequence accession numbers

The nucleotide sequences of the *Alkalibacterium* sp. SL3 16S rDNA and esterase gene (*estSL3*) were deposited into the GenBank database under accession number KT225465 and KT225466, respectively.

## Additional Information

**How to cite this article**: Wang, G. *et al.* A novel cold-adapted and highly salt-tolerant esterase from *Alkalibacterium* sp. SL3 from the sediment of a soda lake. *Sci. Rep.* **6**, 19494; doi: 10.1038/srep19494 (2016).

## Supplementary Material

Supplementary Information

## Figures and Tables

**Table 1:** Effects of metal ions and chemical reagents on rEstSL3 activity.

Chemicals	Relative activity (%)^a^		Chemicals	Relative activity (%)^a^	
	1 mM	5 mM		1 mM	5 mM
Control	100.0 ± 1.6	100 ± 2.1	Co^2+^	87.1 ± 0.3	63.3 ± 1.0
Li^+^	133.9 ± 5.4	103.5 ± 2.5	Cr^3+^	84.9 ± 1.9	42.7 ± 2.0
Na^+^	130.6 ± 5.6	112.0 ± 4.1	Zn^2+^	82.9 ± 2.6	69.0 ± 4.0
K^+^	114.2 ± 0.3	104.1 ± 0.9	Mn^2+^	78.4 ± 5.2	56.4 ± 1.3
Mg^2+^	119.0 ± 2.1	97.6 ± 0.6	Cu^2+^	73.0 ± 2.7	58.0 ± 0.6
Ca^2+^	122.3 ± 1.7	108.3 ± 0.8	Ag^+^	61.5 ± 2.0	25.6 ± 3.3
EDTA	122.1 ± 0.6	112.3 ± 1.8	Fe^2+^	57.6 ± 2.7	39.4 ± 3.8
β-Mercaptalethonal	126.0 ± 0.4	114.6 ± 2.9	Hg^2+^	55.5 ± 1.9	32.1 ± 1.6
Ni^2+^	97.6 ± 0.3	93.2 ± 0.5	Pb^2+^	50.6 ± 0.9	22.8 ± 2.0

^a^Relative activity is defined as the activity percentage against that of control (n = 3).

**Table 2:** Effects of detergents and solvents on rEstSL3 activity.

Chemicals	Concentration (%)	Relative activity (%)^a^	Chemicals	Concentration (%)	Relative activity (%)
Control	0	100.0 ± 2.1			
Tween 20	1	87.0 ± 0.3	Butanol	10	38.4 ± 4.5
	5	39.7 ± 2.8		20	24.3 ± 5.4
	10	25.4 ± 7.0	Isobutanol	10	73.5 ± 0.6
Tween 80	1	131.1 ± 1.4		20	33.0 ± 6.5
	5	121.6 ± 0.8	Isoamyl	10	34.8 ± 2.6
	10	70.8 ± 4.3		20	7.3 ± 3.7
Triton X-100	1	111.6 ± 4.0	Acetone	10	48.4 ± 0.5
	5	76.0 ± 1.4		20	24.4 ± 1.1
	10	35.5 ± 2.9	*n*-Hexane	10	98.1 ± 0.5
SDS	1	68.5 ± 1.2		20	89.1 ± 0.5
	5	26.0 ± 4.4	Glycerol	10	77.5 ± 0.4
	10	ND^b^		20	81.9 ± 0.4
Methanol	10	75.8 ± 0.4	Chloroform	10	57.1 ± 0.6
	20	72.7 ± 2.4		20	45.6 ± 0.5
Ethanol	10	93.9 ± 0.2	Acetonitrile	10	92.7 ± 0.9
	20	87.4 ± 0.2		20	74.2 ± 0.4
Propanol	10	97.9 ± 0.4			
	20	91.2 ± 6.2			

^a^Relative activity is defined as the activity percentage against that of control (n = 3).

^b^ND, not detected.

**Table 3:** Factors affecting the stability and flexibility of EstSL3 and other cold active mesophilic or thermophilic counterparts.

Parameters	EstA	Axe2	LipPc	Est97	EstSL3
PDB code	3DOH	3W7V	4NS4	4AO6	–
T_opt_ (°C)	95	50–60	25	35	30
Gly percent (%)	6.84	8.68	6.31	13.13	8.53
Pro percent (%)	6.84	4.11	4.73	4.63	3.32
Arg/(Arg+ Lys)	15/39	15/35	8/32	15/27	18/29
Number of salt bridges (<3.2 Å)	13	13	9	11	6
Number of hydrogen bonds	1025	973	607	487	126
Hydrogen bond percentage (%)	32.99	52.81	28.14	27.24	7.57
References	32	31	22	27	This study

**Table 4:** Comparison of the amino acid compositions of EstSL3 and other salt-tolerant esterases.

Esterases	Composition (%)		Relative activity (%) with NaCl (M)	References
	Acidic amino acids	Hydrophobic amino acids		
EstSL3	16.6	33.7	102.9 (2)	This study
EstKT7	11.7	38.9	170 (0.5)	42
Est9X	8.2	38.1	190 (4)	43
LipC	16.8	34.9	− (3.4)	44
Est10	11.21	37.7	140 (2)	23
EstPc	11.4	37.9	183.4 (1.5)	22

## References

[b1] Anthonsen H. W. *et al.* Lipases and esterases: a review of their sequences, structure and evolution. Biotechnol Annu Rev 1, 315–371 (1995). 9704093 10.1016/s1387-2656(08)70056-5

[b2] Bornscheuer U. T. Microbial carboxyl esterases: classification, properties and application in biocatalysis. FEMS Microbiol Rev 26, 73–81 (2002). 12007643 10.1111/j.1574-6976.2002.tb00599.x

[b3] Nardini M. & Dijkstra B. W. Alpha/beta hydrolase fold enzymes: the family keeps growing. Curr Opin Struct Biol 9, 732–737 (1999). 10607665 10.1016/s0959-440x(99)00037-8

[b4] Chahinian H. & Sarda L. Distinction between esterases and lipases: comparative biochemical properties of sequence-related carboxylesterases. Protein Pept Lett 16, 1149–1161 (2009). 19508178 10.2174/092986609789071333

[b5] Arpigny J. L. & Jaeger K. E. Bacterial lipolytic enzymes: classification and properties. Biochem J 343, 177–183 (1999). 10493927 PMC1220539

[b6] Akoh C. C., Lee G. C., Liaw Y. C., Huang T. H. & Shaw J. F. GDSL family of serine esterases/lipases. Prog Lipid Res 43, 534–552 (2004). 15522763 10.1016/j.plipres.2004.09.002

[b7] Panda T. & Gowrishankar B. S. Production and applications of esterases. Appl Microbiol Biotechnol 67, 160–169 (2005). 15630579 10.1007/s00253-004-1840-y

[b8] Fucinos P. *et al.* Lipases and esterases from extremophiles: overview and case example of the production and purification of an esterase from Thermus thermophilus HB27. Methods Mol Biol 861, 239–266 (2012). 22426723 10.1007/978-1-61779-600-5_15

[b9] Montella I. R., Schama R. & Valle D. The classification of esterases: an important gene family involved in insecticide resistance–a review. Mem Inst Oswaldo Cruz 107, 437–449 (2012). 22666852 10.1590/s0074-02762012000400001

[b10] Lopez-Lopez O., Cerdan M. E. & Gonzalez Siso M. I. New extremophilic lipases and esterases from metagenomics. Curr Protein Pept Sci 15, 445–455 (2014). 24588890 10.2174/1389203715666140228153801 PMC4093774

[b11] Jones B. E., Grant W. D., Duckworth A. W. & Owenson G. G. Microbial diversity of soda lakes. Extremophiles 2, 191–200 (1998). 9783165 10.1007/s007920050060

[b12] Antony C. P. *et al.* Microbiology of Lonar Lake and other soda lakes. ISME J 7, 468–476 (2013). 23178675 10.1038/ismej.2012.137 PMC3578565

[b13] Sheridan C. Kenyan dispute illuminates bioprospecting difficulties. Nat Biotechnol 22, 1337 (2004). 15529141 10.1038/nbt1104-1337

[b14] Grant W. D. & Heaphy S. Metagenomics and recovery of enzyme genes from alkaline saline environments. Environ Technol 31, 1135–1143 (2010). 20718296 10.1080/09593331003646661

[b15] Rees H. C., Grant S., Jones B., Grant W. D. & Heaphy S. Detecting cellulase and esterase enzyme activities encoded by novel genes present in environmental DNA libraries. Extremophiles 7, 415–421 (2003). 12845554 10.1007/s00792-003-0339-2

[b16] Ghasemi Y. *et al.* Isolation and characterization of some moderately halophilic bacteria with lipase activity. Mikrobiologiia 80, 477–481 (2011). 22073547

[b17] Shen J., Cao J. T. & Wu Y. H. Paleoclimatic changes in Dabusu Lake. Chin J Oceanol Limnol 19, 91–96 (2001).

[b18] Wang G., Huang X., Ng T. B., Lin J. & Ye X. Y. High phylogenetic diversity of glycosyl hydrolase family 10 and 11 xylanases in the sediment of Lake Dabusu in China. PLoS ONE 9, e112798 (2014). 25392912 10.1371/journal.pone.0112798 PMC4231106

[b19] Huang X., Lin J., Ye X. & Wang G. Molecular Characterization of a Thermophilic and Salt- and Alkaline-Tolerant Xylanase from *Planococcus* sp. SL4, a Strain Isolated from the Sediment of a Soda Lake. J Microbiol Biotechnol 25, 662–671 (2015). 25381738 10.4014/jmb.1408.08062

[b20] Tutino M. L., di Prisco G., Marino G. & de Pascale D. Cold-adapted esterases and lipases: from fundamentals to application. Protein Pept Lett 16, 1172–1180 (2009). 19508185 10.2174/092986609789071270

[b21] Joseph B., Ramteke P. W. & Thomas G. Cold active microbial lipases: some hot issues and recent developments. Biotechnol Adv 26, 457–470, doi: 10.1016/j.biotechadv.2008.05.003 (2008). 18571355

[b22] Novototskaya-Vlasova K., Petrovskaya L., Yakimov S. & Gilichinsky D. Cloning, purification, and characterization of a cold-adapted esterase produced by *Psychrobacter cryohalolentis* K5T from Siberian cryopeg. FEMS Microbiol Ecol 82, 367–375 (2012). 22486752 10.1111/j.1574-6941.2012.01385.x

[b23] Wu G., Zhan T., Shao Z. & Liu Z. Characterization of a cold-adapted and salt-tolerant esterase from a psychrotrophic bacterium *Psychrobacter pacificensis*. Extremophiles 17, 809–819 (2013). 23868329 10.1007/s00792-013-0562-4

[b24] Wu G. *et al.* A cold-adapted, solvent and salt tolerant esterase from marine bacterium Psychrobacter pacificensis. Int J Biol Macromol 81, 180–187 (2015). 26231332 10.1016/j.ijbiomac.2015.07.045

[b25] Zhang S., Wu G., Liu Z. & Shao Z. Characterization of EstB, a novel cold-active and organic solvent-tolerant esterase from marine microorganism *Alcanivorax dieselolei* B-5(T). Extremophiles 18, 251–259 (2014). 24318107 10.1007/s00792-013-0612-y

[b26] Fu C. *et al.* Molecular cloning and characterization of a new cold-active esterase from a deep-sea metagenomic library. Appl Microbiol Biotechnol 90, 961–970 (2011). 21336688 10.1007/s00253-010-3079-0

[b27] Fu J. *et al.* Functional and structural studies of a novel cold-adapted esterase from an Arctic intertidal metagenomic library. Appl Microbiol Biotechnol 97, 3965–3978 (2013). 22832985 10.1007/s00253-012-4276-9

[b28] Hu X. P., Heath C., Taylor M. P., Tuffin M. & Cowan D. A novel, extremely alkaliphilic and cold-active esterase from Antarctic desert soil. Extremophiles 16, 79–86 (2012). 22052561 10.1007/s00792-011-0407-y

[b29] Seo S. *et al.* Characterization of a novel cold-active esterase isolated from swamp sediment metagenome. World J Microbiol Biotechnol 30, 879–886 (2014). 24353039 10.1007/s11274-013-1496-9

[b30] Esteban-Torres M., Mancheno J. M., de las Rivas B. & Munoz R. Characterization of a cold-active esterase from *Lactobacillus plantarum* suitable for food fermentations. J Agric Food Chem 62, 5126–5132 (2014). 24856291 10.1021/jf501493z

[b31] Lansky S. *et al.* Crystallization and preliminary crystallographic analysis of Axe2, an acetylxylan esterase from *Geobacillus stearothermophilus*. Acta Crystallogr Sect F Struct Biol Cryst Commun 69, 430–434 (2013). 10.1107/S1744309113004260 PMC3614171 23545652

[b32] Levisson M. *et al.* Crystal structure and biochemical properties of a novel thermostable esterase containing an immunoglobulin-like domain. J Mol Biol 385, 949–962 (2009). 19013466 10.1016/j.jmb.2008.10.075

[b33] Karan R., Capes M. D. & Dassarma S. Function and biotechnology of extremophilic enzymes in low water activity. Aquat Biosyst 8, 4 (2012). 22480329 10.1186/2046-9063-8-4 PMC3310334

[b34] Liu Y. G. & Whittier R. F. Thermal asymmetric interlaced PCR: automatable amplification and sequencing of insert end fragments from P1 and YAC clones for chromosome walking. Genomics 25, 674–681 (1995). 7759102 10.1016/0888-7543(95)80010-j

[b35] Tamura K., Dudley J., Nei M. & Kumar S. MEGA4: Molecular Evolutionary Genetics Analysis (MEGA) software version 4.0. Mol Biol Evol 24, 1596–1599 (2007). 17488738 10.1093/molbev/msm092

[b36] Yang J. *et al.* The I-TASSER Suite: protein structure and function prediction. Nat Methods 12, 7–8 (2015). 25549265 10.1038/nmeth.3213 PMC4428668

[b37] Mariani V., Kiefer F., Schmidt T., Haas J. & Schwede T. Assessment of template based protein structure predictions in CASP9. Proteins 79, 37–58 (2011). 22002823 10.1002/prot.23177

[b38] Xu J. & Zhang Y. How significant is a protein structure similarity with TM-score = 0.5? Bioinformatics 26, 889–895 (2010). 20164152 10.1093/bioinformatics/btq066 PMC2913670

[b39] Zhou J. *et al.* Molecular and biochemical characterization of a novel xylanase from the symbiotic *Sphingobacterium* sp. TN19. Appl Microbiol Biotechnol 85, 323–333 (2009). 19554324 10.1007/s00253-009-2081-x

[b40] Bradford M. M. A rapid and sensitive method for the quantitation of microgram quantities of protein utilizing the principle of protein-dye binding. Anal Biochem 72, 248–254 (1976). 942051 10.1016/0003-2697(76)90527-3

[b41] Manco G., Di Gennaro S., De Rosa M. & Rossi M. Purification and characterization of a thermostable carboxylesterase from the thermoacidophilic eubacterium *Bacillus acidocaldarius*. Eur J Biochem 221, 965–972 (1994). 8181479 10.1111/j.1432-1033.1994.tb18812.x

[b42] Jeon J. H. *et al.* Identification of a new subfamily of salt-tolerant esterases from a metagenomic library of tidal flat sediment. Appl Microbiol Biotechnol 93, 623–631 (2012). 21720822 10.1007/s00253-011-3433-x

[b43] Fang Z. *et al.* A novel esterase from a marine metagenomic library exhibiting salt tolerance ability. J Microbiol Biotechnol 24, 771–780 (2014). 24633233 10.4014/jmb.1311.11071

[b44] Rao L. *et al.* Solution behavior and activity of a halophilic esterase under high salt concentration. PLoS ONE 4, e6980 (2009). 19759821 10.1371/journal.pone.0006980 PMC2736375

