# Supplementary material for: A novel cold-adapted and highly salt-tolerant esterase from Alkalibacterium sp. SL3 from the sediment of a soda lake
Source: Sci Rep. 2016 Feb 26;6:19494. doi: 10.1038/srep19494 (PMC4768246; doi:10.1038/srep19494)
Supplement: Supplementary Information [file srep19494-s1.pdf]

Supporting Information to

A novel cold-adapted and highly salt-tolerant esterase from *Alkalibacterium* sp. SL3 from the sediment of a soda lake

Guozeng Wang[a, b§] · Qiaohuang Wang[a§] · Xianju Lin[a] · Tzi Bun Ng[c] · Renxiang Yan[a] ·Juan Lin[a, b*] ·Xiuyun Ye[a, b*]

[a] College of Biological Science and Engineering, Fuzhou University, Fuzhou 350108, P. R. China

[b] Fujian Key Laboratory of Marine Enzyme Engineering, Fuzhou 350002, P.R. China

[c] School of Biomedical Sciences, Faculty of Medicine, The Chinese University of Hong Kong, Hong Kong, China

[*]Corresponding authors: xiuyunye@fzu.edu.cn, ljuan@fzu.edu.cn

Fig.S1

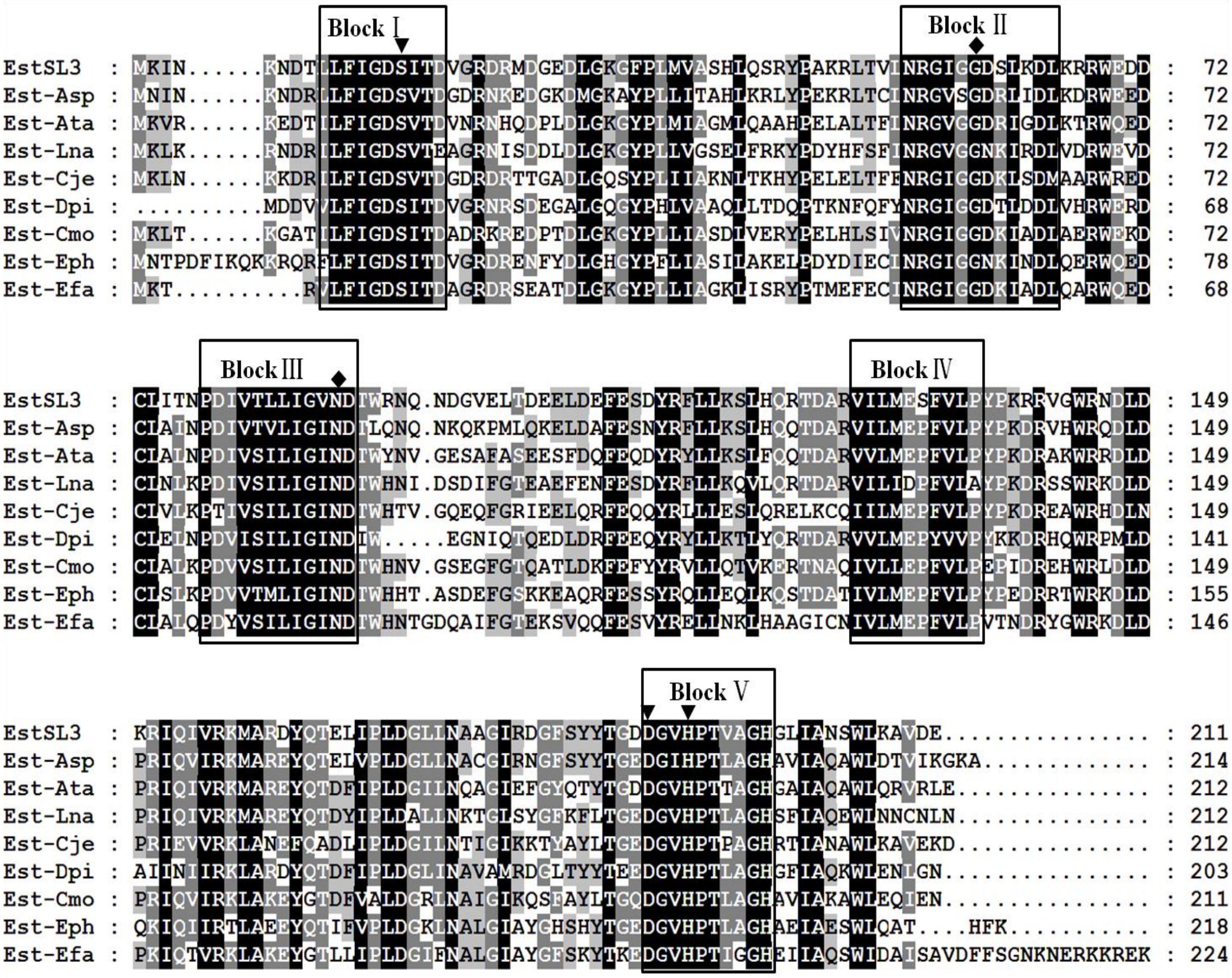

Supplementary Fig. S1 Multiple alignment of EstSL3 and eleven putative esterases from GenBank. Identical and similar amino acids are highlighted in solid black and grey, respectively. Five consensus sequence blocks (I–V) are boxed. Putative catalytic triad of Ser^{15}, Asp^{189}, and His^{192} are marked with solid triangle. Gly^{59} and Asn^{88} involved in the oxyanion hole formation are marked with solid diamond. Sequence names, microbial sources and GenBank accession numbers are given as follows: Est-Asp: *Alkalibacterium* sp. AK22 (WP_034300718); Est-Ata: *Atopococcus tabaci* (WP_028274330); Est-Lna: *Lacticigenium naphtae* (WP_035618581); Est-Cje: *Carnobacterium jeotgali* (WP_035050588); Est-Dpi: *Dolosigranulum pigrum* (WP_040376845); Est-Cmo: *Carnobacterium mobile* (WP_035032144); Est-Eph: *Enterococcus phoeniculicola* ATCC BAA-412 (EOL42081); Est-Efa: *Enterococcus faecium* (WP_002345223); and EstSL3: *Alkalibacterium* sp. SL3 (KT225466).

Fig.S2

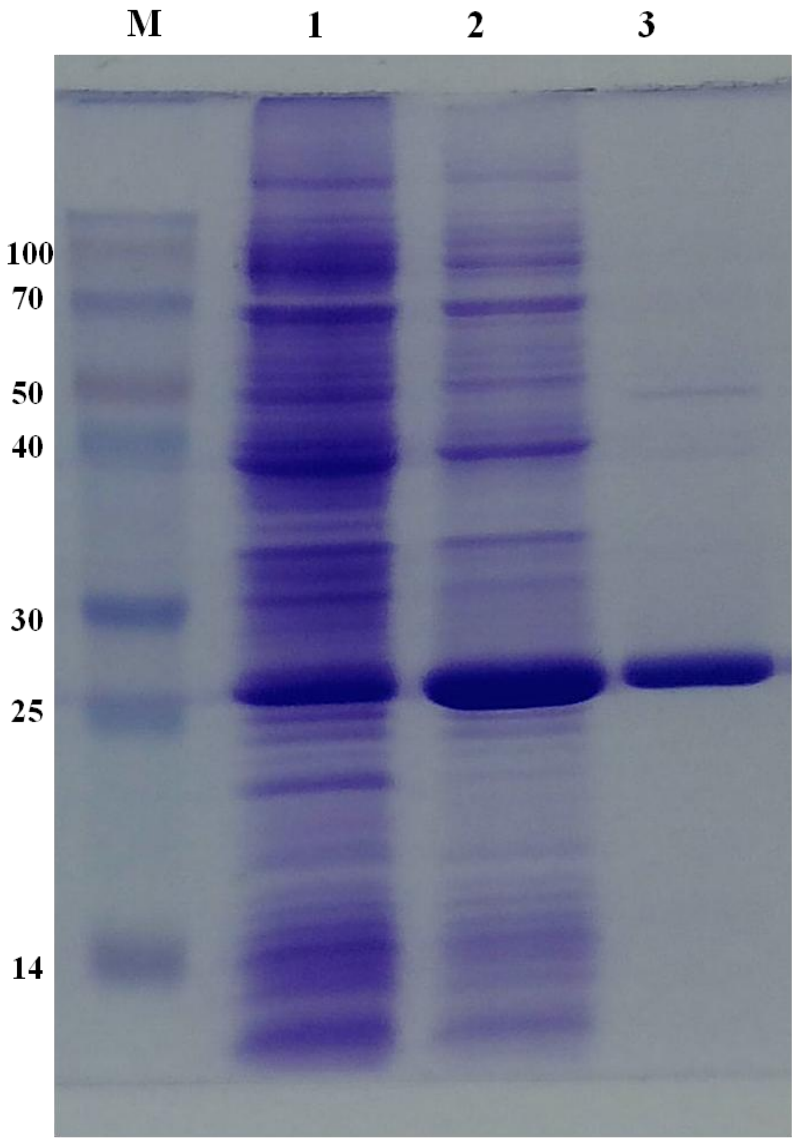

Supplementary Fig. S2 SDS-PAGE analysis of purified rEstSL3. Lanes: M, the protein marker, 1, the cell extract of an uninduced transformant harboring pET-*estSL3*; 2, the cell extract of an induced transformant harboring pET-*estSL3*; 3, the purified rEstSL3 after Ni-affinity chromatography.

Table S1 Primers used in this study

**Primer name**	**Primer sequence (5'→3')[a]**	**Size**
27F	AGAGTTTGATCCTGGCTCAG	20
1492R	GGTTACCTTGTTACGACTT	19
estSL3-uSP1	CAGCCCACTCTTCTTTTCGGATAAGGCAG	29
estSL3-uSP2	GACTCGAGCATCCGTTCTCTGGTGAAG	27
estSL3-uSP3	CCTCATCTGTAAGTTCTACTCCGTCATTCTG	31
estSL3-m-F	GCGCCATGGGCATGAAAATCAACAAGAATGATACG	35
estSL3-m-R	CCGAAGCTTTTCATCGACAGCTTTCAGCC	29

[a]Restriction sites are underlined.
